# Enhanced lung volumes and pressures in traditional wind instrument musicians: The case of the Mejwez

**DOI:** 10.1371/journal.pone.0344473

**Published:** 2026-03-05

**Authors:** Mohammad Z. Darabseh, Aseel Aburub, Ragad Alkaraki, Abdullah Saber Alshammari, Yazan Almansi, Ghaith Dalalah, Csaba Melczer, Pongrác Ács

**Affiliations:** 1 Department of Physiotherapy, School of Rehabilitation Sciences, The University of Jordan, Amman, Jordan; 2 Department of Physiotherapy, Faculty of Allied Medical Sciences, Applied Science Private University, Amman, Jordan; 3 Department of Allied Medical Sciences, Faculty of Applied Medical Sciences, Jordan University of Science and Technology, Al-Ramtha, Jordan; 4 Department of Community and Mental Health, College of Nursing, Dubai Medical University, Dubai, United Arab Emirates; 5 Institute of Physiotherapy and Sports Science, Faculty of Health Sciences, University of Pécs, Pécs, Hungary; 6 Physical Activity Research Group, János Szentágothai Research Center, University of Pécs, Pécs, Hungary; Faculty of Medicine Universitas Indonesia - CIPTO Mangunkusumo General Hospital, INDONESIA

## Abstract

To evaluate pulmonary function and respiratory muscle strength in Mejwez players compared to predicted norms, and to assess the influence of smoking behaviours on these outcomes. A cross-sectional study was conducted involving adult Mejwez players. Spirometric parameters (forced expiratory volume in one second (FEV₁), forced vital capacity (FVC), and FEV₁/FVC) and respiratory pressures (maximal inspiratory pressure (MIP) and maximal expiratory pressure (MEP)) were measured and compared to predicted values. Multiple linear regression was used to assess the impact of smoking behaviours on respiratory outcomes. Mejwez players demonstrated significantly higher FEV₁ (+0.38 L, p < 0.001), FVC (+0.24 L, p = 0.001), MIP (+7.7 cm H₂O, p = 0.014), and MEP (+7.1 cm H₂O, p = 0.017) compared to predicted norms. Waterpipe smoking was significantly associated with reduced MIP (β = –9.3, p = 0.024) and MEP (β = –8.6, p = 0.025), while cigarette and vaping use showed no significant associations. This is the first study to characterise respiratory adaptations in Mejwez players, revealing enhanced pulmonary and respiratory muscle function. Waterpipe smoking was a significant negative predictor of respiratory strength. These findings highlight traditional music as a culturally relevant tool for respiratory health promotion and underscore the need for interventions addressing waterpipe use in the Middle East.

## Introduction

Pulmonary function and respiratory muscle strength are critical determinants of respiratory health, influencing exercise capacity, airway clearance, and overall quality of life. Spirometric parameters such as forced expiratory volume in one second (FEV₁) and forced vital capacity (FVC), along with maximal inspiratory (MIP) and expiratory pressures (MEP), serve as key indicators of ventilatory efficiency and muscular effort [1,2]. While these metrics are commonly used in clinical and research settings to assess disease burden and therapeutic response, emerging evidence suggests that certain lifestyle practices, particularly musical activities, may positively modulate respiratory performance.

Wind instrument playing has garnered attention for its potential to enhance pulmonary function through repetitive and controlled breathing manoeuvres. Several studies have reported increased vital capacity, improved respiratory pressures, and enhanced airway control among professional musicians [3–5]. These adaptations are thought to result from the sustained respiratory effort required to produce sound, particularly in instruments that demand circular breathing or high intraoral pressure. However, the existing literature is predominantly centred on Western instruments such as the trumpet, clarinet, and saxophone, with limited exploration of traditional wind instruments practised in non-Western regions.

The Mejwez is a double-pipe reed instrument widely played across the Middle East region. It is characterised by continuous airflow and circular breathing, requiring players to maintain uninterrupted sound production while simultaneously inhaling through the nose and exhaling through the mouth. Despite its cultural prominence and unique respiratory demands, no studies to date have investigated the physiological impact of Mejwez playing on lung function or respiratory muscle strength. This represents a significant gap in the literature, particularly given the growing interest in culturally tailored, non-pharmacological interventions for respiratory health.

In parallel, smoking behaviours, especially waterpipe use, remain prevalent in the Middle East and pose substantial risks to pulmonary function. Waterpipe smoking has been associated with reduced lung volumes, impaired gas exchange, and diminished respiratory muscle strength due to prolonged inhalation and exposure to high concentrations of carbon monoxide and particulate matter [6,7]. While cigarette smoking and vaping have been extensively studied, the specific impact of waterpipe use on respiratory pressures remains underexplored, particularly in populations engaged in high-respiratory demand activities such as wind instrument playing.

Given these gaps, the present study aimed to: (1) evaluate pulmonary function and respiratory muscle strength in Mejwez players compared to predicted norms; and (2) assess the impact of smoking behaviours, including cigarette, vaping, and waterpipe use, on these respiratory parameters. By focusing on a culturally significant instrument and regionally relevant exposures, this study offers novel insights into the intersection of music, lifestyle, and respiratory physiology. This study findings might contribute to a more inclusive understanding of how traditional practices may influence health and inform future interventions tailored to Middle Eastern populations.

## Methods

### Study design and participants

This cross-sectional observational study was conducted to evaluate pulmonary function and respiratory muscle strength in a cohort of adult males. A total of 41 participants were recruited from the general population in Jordan. Inclusion criteria were: male sex, age between 18 and 60 years, and absence of acute respiratory illness at the time of testing. Participants with known chronic pulmonary diseases (e.g., COPD, asthma), cardiovascular conditions, or neuromuscular disorders were excluded.

Ethical approval was obtained from the Research Ethics Committee at the Faculty of Allied Medical Sciences at Applied Science Private University (Approval number: AMS-2024–4). All subjects provided informed consent prior to participation, and the procedures adhered to the ethical standards of the Declaration of Helsinki.

### Demographic and behavioural data collection

Demographic data including age, height, weight, and body mass index (BMI), were recorded. Smoking status was categorised as current smoker, non-smoker, or ex-smoker. Vaping and waterpipe use were similarly classified. Behavioural data were self-reported using a structured questionnaire administered prior to spirometry testing.

### Pulmonary function testing

Spirometry was performed using a calibrated digital spirometer (BTL Spirometry, Hertfordshire, United Kingdom) following European Respiratory Society/American Thoracic Society (ERS/ATS) guidelines [8]. Each participant wore a nose clip and performed a minimum of three acceptable manoeuvres, with at least 1–2 minutes of rest between attempts. Manoeuvres were excluded if the participant prematurely stopped exhalation, coughed during the first second, failed to fully seal lips around the mouthpiece, obstructed the mouthpiece with teeth or tongue, or if the effort appeared submaximal. The testing session was concluded when the largest two FEV₁ and the largest two FVC values were each within 0.15 L of each other across at least three manoeuvres [9]. Each participant performed a minimum of three acceptable manoeuvres, and the highest values were used for analysis. Parameters measured included: Forced Expiratory Volume in 1 second (FEV₁); Forced Vital Capacity (FVC) and FEV₁/FVC ratio. Predicted values for FEV₁ and FVC were calculated based on age, height, and ethnicity using standardised reference equations. Each participant performed a minimum of three acceptable manoeuvres, and the highest values were used for analysis.

### Respiratory muscle strength assessment

Maximal Inspiratory Pressure (MIP) and Maximal Expiratory Pressure (MEP) were measured using a handheld respiratory pressure meter (Contec RPM10 Respiratory Pressure Meter, Qinhuangdao, China). Participants were instructed to perform maximal inspiratory and expiratory efforts against an occluded airway following ATS/ERS recommendations [2]. For all manoeuvres, attempts were repeated with a 30-second interval between each effort to minimise respiratory muscle fatigue, until a maximum value was achieved. Predicted values for MIP and MEP were derived from age- and sex-specific reference equations.

### Statistical analysis

Descriptive statistics were computed for all variables. Paired sample t-tests were used to compare measured vs predicted values for FEV₁, FVC, MIP, and MEP. Multiple linear regression analyses were conducted to assess the impact of smoking behaviours (cigarette smoking, vaping, waterpipe use) on spirometry outcomes and respiratory pressures. Predictor variables were coded as binary (yes/no) for regression modelling. Statistical significance was set at p < 0.05. All analyses were performed using SPSS version 26.0 (IBM Corp., Armonk, NY).

## Results

### Participant characteristics

A total of 41 adult male participants were included in the analysis. The mean age was 35.6 ± 10.5 years, with a mean height of 172.1 ± 7.4 cm and a mean weight of 78.1 ± 16.3 kg. The average body mass index (BMI) was 26.3 ± 4.8 kg/m², with 32% classified as normal weight, 46% as overweight, and 22% as obese. Regarding smoking behaviours, 56% were current smokers, 34% were non-smokers, and 10% were ex-smokers. Vaping was reported by 5% of participants, while 10% were current waterpipe users (Table 1).

**Table 1:** Demographic characteristics of study participants (N = 41).

Variable	Category/ Statistic	Value
Sex	Male	100% (41/41)
Age (years)	Mean ± SD	34.95 ± 10.5
Height (cm)	Mean ± SD	172.8 ± 7.4
Weight (kg)	Mean ± SD	78.5 ± 16.3
BMI (kg/m²)	Mean ± SD	26.3 ± 4.8

SD: Standard deviation.

### Comparison of measured vs predicted spirometry values

Measured spirometry values were significantly higher than predicted reference values. The mean FEV₁ was 4.29 ± 0.63 L compared to a predicted mean of 3.91 ± 0.47 L (t = 5.94, p < 0.001). Similarly, the mean FVC was 4.87 ± 0.66 L versus a predicted 4.63 ± 0.43 L (t = 3.52, p = 0.001). These findings suggest that participants exhibited above-average pulmonary function relative to standardised norms. The FEV₁/FVC ratio averaged 87.3 ± 6.6%, consistent with normal ventilatory patterns (Table 2).

**Table 2:** Comparison of measured vs predicted spirometry parameters.

Parameter	Measured Mean ± SD	Predicted Mean ± SD	Mean Difference ± SD	t-value	p-value	Interpretation
FEV₁ (L)	4.29 ± 0.63	3.91 ± 0.47	+0.38 ± 0.49	5.94	<0.001	Significant ↑
FVC (L)	4.87 ± 0.66	4.63 ± 0.43	+0.24 ± 0.52	3.52	0.001	Significant ↑
FEV₁/FVC (%)	87.3 ± 6.6	—	—	—	—	Not applicable

FEV₁: Forced Expiratory Volume in 1 second; FVC: Forced Vital Capacity; SD: Standard Deviation; ↑ = Higher than predicted.

### Respiratory muscle strength

Measured respiratory pressures also exceeded predicted values. The mean MIP was 112.7 ± 26.3 cm H₂O, significantly higher than the predicted 105.0 ± 22.5 cm H₂O (t = 2.56, p = 0.014). MEP values followed a similar trend, with a measured mean of 137.1 ± 22.7 cm H₂O compared to a predicted 130.0 ± 20.1 cm H₂O (t = 2.49, p = 0.017) (Table 3).

**Table 3:** Comparison of measured vs predicted respiratory pressures.

Parameter	Measured Mean ± SD	Predicted Mean ± SD	Mean Difference ± SD	t-value	p-value	Interpretation
MIP (cm H₂O)	112.7 ± 26.3	105.0 ± 22.5	+7.7 ± 18.4	2.56	0.014	Significant ↑
MEP (cm H₂O)	137.1 ± 22.7	130.0 ± 20.1	+7.1 ± 17.2	2.49	0.017	Significant ↑

MIP: Maximal Inspiratory Pressure; MEP: Maximal Expiratory Pressure; SD: Standard Deviation; ↑ = Higher than predicted.

### Regression analysis of smoking behaviours

Multiple linear regression analyses were conducted to assess the impact of smoking, vaping, and waterpipe use on spirometry and respiratory pressure outcomes. Waterpipe use was negatively associated with both MIP (β = −9.3, p = 0.024) and MEP (β = −8.6, p = 0.025), indicating a statistically significant reduction in respiratory muscle strength among current users. Marginal negative associations were observed between waterpipe use and FEV₁ (β = −0.15, p = 0.067) and FVC (β = −0.18, p = 0.052). No significant associations were found for cigarette smoking or vaping across any measured outcomes (Table 4).

**Table 4:** Multiple regression analysis of smoking behaviours on spirometry outcomes.

Dependent Variable	Predictor(s)	β Coefficient	Std. Error	t-value	p-value	Interpretation
FEV₁ (L)	Smoking (yes vs no)	−0.12	0.09	−1.33	0.19	NS
	Vaping (yes vs no)	−0.08	0.10	−0.80	0.43	NS
	Waterpipe (yes vs no)	−0.15	0.08	−1.88	0.067	Marginal ↓
FVC (L)	Smoking	−0.10	0.10	−1.00	0.32	NS
	Vaping	−0.05	0.11	−0.45	0.65	NS
	Waterpipe	−0.18	0.09	−2.00	0.052	Marginal ↓
MIP (cm H₂O)	Smoking	−6.5	4.2	−1.55	0.13	NS
	Vaping	−4.1	4.5	−0.91	0.37	NS
	Waterpipe	−9.3	4.0	−2.33	0.024	Significant ↓
MEP (cm H₂O)	Smoking	−5.8	3.9	−1.49	0.14	NS
	Vaping	−3.7	4.2	−0.88	0.38	NS
	Waterpipe	−8.6	3.7	−2.32	0.025	Significant ↓

FEV₁: Forced Expiratory Volume in 1 second; FVC: Forced Vital Capacity; MIP: Maximal Inspiratory Pressure; MEP: Maximal Expiratory Pressure; NS: Not Significant; ↓ = Negative association.

## Discussion

This study provides novel insights into the pulmonary and respiratory muscle function of individuals who play the Mejwez, a traditional Middle Eastern wind instrument. To our knowledge, this is the first investigation to assess lung volumes and respiratory pressures in Mejwez players, a population previously unrepresented in the literature. These findings demonstrate significantly higher measured values of FEV₁, FVC, MIP, and MEP compared to predicted norms, suggesting enhanced respiratory performance among these musicians. These results align with and extend prior research on wind instrument players, while introducing a culturally specific context with potential clinical implications.

Participants exhibited significantly elevated FEV₁ and FVC values, with mean differences of +0.38 L and +0.24 L, respectively, both reaching statistical significance (p < 0.001 and p = 0.001). Respiratory muscle strength, as measured by MIP and MEP, was also significantly higher than predicted (+7.7 cm H₂O and +7.1 cm H₂O, respectively). These enhancements suggest that regular Mejwez playing may confer physiological benefits similar to those observed in Western wind instrument players, such as trumpeters, clarinettists, and oboists [3,10].

Unlike previous studies, which have focused on Western instruments, this research centres on the Mejwez, a double-pipe reed instrument requiring continuous circular breathing and high intraoral pressure. Its unique playing technique may demand greater respiratory effort and control, potentially leading to superior pulmonary adaptation. The cultural specificity of this instrument, widely played across the Middle East, adds a novel dimension to the global understanding of music-related respiratory physiology.

Prior studies have consistently shown that wind instrument players possess enhanced lung function compared to non-players [4,5]. For instance, a study conducted among professional vocalists and wind instrumentalists reported increased vital capacity and expiratory pressures [4]. Similarly, a previous study reported that long-term saxophone and trumpet players had significantly higher MIP and MEP values than matched controls [5]. These findings corroborate these trends and suggest that Mejwez playing may elicit comparable or even greater respiratory muscle engagement due to its continuous airflow demands.

However, another study has reported mixed results [10]. The study found no significant differences in spirometry between wind instrument players and controls, attributing variability to instrument type and playing intensity [10]. This study addresses this gap by focusing on a single instrument with a consistent technique, thereby reducing heterogeneity and strengthening the observed associations.

The elevated respiratory parameters observed in Mejwez players may have important clinical implications. Enhanced MIP and MEP are associated with improved cough efficacy, reduced risk of respiratory complications, and better outcomes in pulmonary rehabilitation [2]. These findings suggest that Mejwez playing could be explored as a culturally relevant adjunct to respiratory therapy, particularly in Middle Eastern populations.

Moreover, the study contributes to the growing body of evidence supporting music-based interventions for respiratory health. Wind instrument training has been proposed as a non-pharmacological strategy for improving lung function in patients with asthma, COPD, and neuromuscular disorders [5,11]. The Mejwez, with its rhythmic and sustained breathing patterns, may offer similar therapeutic potential.

In addition to the primary findings on Mejwez players, regression analysis revealed nuanced associations between smoking behaviours and respiratory outcomes. Cigarette smoking and vaping did not significantly predict reductions in FEV₁, FVC, MIP, or MEP, with all p-values exceeding 0.13, suggesting no strong independent effect in this cohort. However, waterpipe smoking demonstrated a more pronounced impact, particularly on respiratory muscle strength. Waterpipe use was associated with a significant reduction in MIP (β = –9.3 cm H₂O, p = 0.024) and MEP (β = –8.6 cm H₂O, p = 0.025), and marginal reductions in FEV₁ and FVC (p = 0.067 and p = 0.052, respectively). These findings align with emerging evidence that waterpipe smoking, despite its cultural normalization, poses substantial risks to pulmonary health due to prolonged inhalation volumes and high carbon monoxide exposure [6,7].

Interestingly, the lack of significant associations for cigarette and vaping use may reflect either underreporting, low cumulative exposure, or the protective influence of regular wind instrument training. Previous studies have shown that chronic smoking impairs respiratory muscle strength and lung volumes [12], but our data suggest that Mejwez playing may partially buffer these effects, particularly in individuals with mixed exposure profiles. Nonetheless, the deleterious impact of waterpipe use on respiratory pressures underscores the need for targeted public health messaging in regions where this practice is prevalent.

Despite its strengths, this study has several limitations. Smoking exposure was assessed using self‑reported questionnaires, which are subject to recall bias and underreporting. While such tools are widely used in the smoking literature, they remain imperfect measures of exposure. In addition, the small sample size, reflecting the limited number of Mejwez players and recruitment challenges in rural areas, reduced the statistical power of subgroup analyses. As a result, subgroup comparisons were considered exploratory and were not included in the final study conclusions. Additionally, this study is a cross‑sectional design precludes causal inference, and the absence of a matched non‑wind instrument control group limits the extent to which our findings can be directly compared with a more representative general population. Furthermore, reliance on predicted reference values rather than actual controls constrains the interpretation of differences, which should be considered associations rather than causal effects. Moreover, confounding factors such as physical activity, diet, and environmental exposures were not controlled, which may influence pulmonary outcomes. Furthermore, data on the duration and intensity of Mejwez practice were not obtained, which limits the ability to explore dose-response relationships between practice exposure and respiratory outcomes. Future studies should incorporate these variables to better characterise the impact of practice intensity and duration. Finally, the reliance on predicted norms derived from Western populations may introduce bias when applied to Middle Eastern cohorts.

Future research should explore the longitudinal effects of Mejwez playing on respiratory health, including its impact on disease progression in individuals with chronic lung conditions. Comparative studies across different wind instruments, both traditional and modern, could elucidate the specific biomechanical demands and training effects. Additionally, integrating imaging techniques such as diaphragm ultrasonography or respiratory muscle electromyography may provide deeper insights into the physiological adaptations associated with instrument playing.

Given the cultural significance of the Mejwez, community-based interventions incorporating music and breathing exercises could be developed to promote respiratory wellness in underserved populations. Collaborations between pulmonologists, respiratory physiotherapists, music therapists, and ethnomusicologists may yield innovative, culturally tailored health programs.

## Conclusion

This study is the first to characterise the pulmonary and respiratory muscle function of Mejwez players, revealing significantly enhanced FEV₁, FVC, MIP, and MEP values compared to predicted norms. These findings align with existing literature on wind instrument players and underscore the potential of traditional music practices to influence respiratory physiology. The Mejwez, deeply rooted in Middle Eastern heritage, may serve not only as a cultural artefact but also as a tool for respiratory health promotion. Further research is warranted to explore its therapeutic applications and long-term benefits.

## References

[1] Pellegrino R, Viegi G, Brusasco V, Crapo RO, Burgos F, Casaburi R, et al. Interpretative strategies for lung function tests. Eur Respir J. 2005;26(5):948–68. doi: 10.1183/09031936.05.00035205 16264058

[2] American Thoracic Society/European Respiratory Society. ATS/ERS Statement on respiratory muscle testing. Am J Respir Crit Care Med. 2002;166(4):518–624. doi: 10.1164/rccm.166.4.518 12186831

[3] Bouhuys A. Lung volumes and breathing patterns in wind-instrument players. J Appl Physiol. 1964;19:967–75. doi: 10.1152/jappl.1964.19.5.967 14207753

[4] Nicoară R, Monea D. Respiratory muscle training methods for improving the lung capacity of professional vocalists and wind instrumentists. Studia UBB Educatio Artis Gymn. 2021;66(1):85–100. doi: 10.24193/subbeag.66(1).09

[5] Antoniadou M, Michaelidis V, Tsara V. Lung function in wind instrument players. Pneumon. 2012;25(2).

[6] Aboaziza E, Eissenberg T. Waterpipe tobacco smoking: what is the evidence that it supports nicotine/tobacco dependence? Tob Control. 2015;24 Suppl 1(Suppl 1):i44–53. doi: 10.1136/tobaccocontrol-2014-051910 25492935 PMC4345797

[7] El-Zaatari ZM, Chami HA, Zaatari GS. Health effects associated with waterpipe smoking. Tob Control. 2015;24 Suppl 1(Suppl 1):i31–43. doi: 10.1136/tobaccocontrol-2014-051908 25661414 PMC4345795

[8] Graham BL, Steenbruggen I, Miller MR, Barjaktarevic IZ, Cooper BG, Hall GL, et al. Standardization of spirometry 2019 update. An official American thoracic society and European respiratory society technical statement. Am J Resp Critic Care Med. 2019;200(8):e70–e88. 10.1164/rccm.201908-1590ST PMC6794117 31613151

[9] Miller M, Hankinson J, Brusasco V, Burgos F, Casaburi R, Coates A, et al. Standardisation of spirometry. Eur Respir J. 2005;26(2):319–38. 16055882 10.1183/09031936.05.00034805

[10] Schorr-Lesnick B, Teirstein AS, Brown LK, Miller A. Pulmonary function in singers and wind-instrument players. Chest. 1985;88(2):201–5. doi: 10.1378/chest.88.2.201 4017673

[11] Lötters F, van Tol B, Kwakkel G, Gosselink R. Effects of controlled inspiratory muscle training in patients with COPD: a meta-analysis. Eur Respir J. 2002;20(3):570–6. doi: 10.1183/09031936.02.00237402 12358330

[12] Gan WQ, Man SFP, Senthilselvan A, Sin DD. Association between chronic obstructive pulmonary disease and systemic inflammation: a systematic review and a meta-analysis. Thorax. 2004;59(7):574–80. doi: 10.1136/thx.2003.019588 15223864 PMC1747070

